# Study on low-temperature cycle failure mechanism of a ternary lithium ion battery

**DOI:** 10.1039/d2ra02518c

**Published:** 2022-07-19

**Authors:** Suijun Wang, Chen Hu, Ran Yu, Zhaoqin Sun, Yi Jin

**Affiliations:** State Key Laboratory of Operation and Control of Renewable Energy & Storage Systems, China Electric Power Research Institute Beijing 100192 China wangsuijun@jasolar.com

## Abstract

This study is focused on the changes in parameters such as discharge capacity, and the possible failure mechanism of a 25 Ah ternary lithium ion battery during cycling at −10 °C. A new battery and a battery after 500 cycles were disassembled. The morphology and structure of the cathode and anode electrodes were characterized. Transmission electron microscopy (TEM) and X-ray photoelectron absorption spectroscopy (XPS) were used to analyze the changes in the microstructure and chemical environment of the anode electrode interface. The results show that after 500 cycles at −10 °C, the capacity of the battery is only 18.3 Ah, and there is a large irreversible capacity loss. The battery samples after low-temperature cycling produced gas during storage at 25 °C. It is found that a large amount of lithium plating on the anode surface is an important factor for the reduction in battery capacity. The dissolution of transition metal elements in the cathode electrode and deposition on the anode electrode, and the catalytic decomposition of electrolyte at the anode interface are the main reasons for the gassing of the battery.

## Abstract 2

The failure of low-temperature performance of ternary lithium-ion battery is the result of side reactions such as lithium deposition, positive material transition metal ion dissolution and electrolyte decomposition.

## Graphical abstract

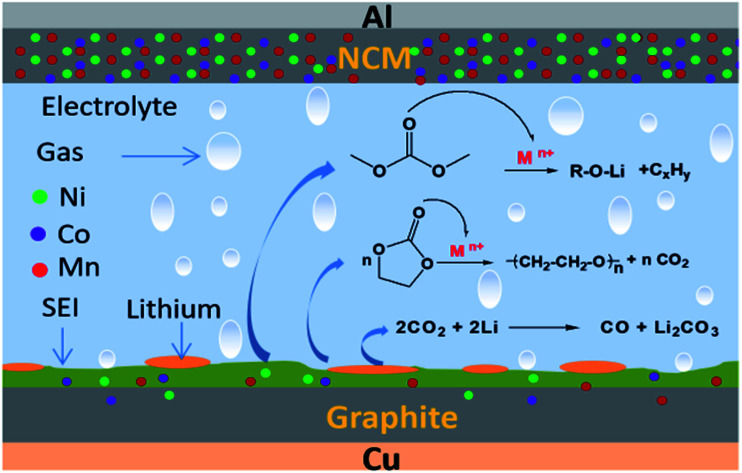

## Introduction

The severe challenges of energy shortage and greenhouse gas emissions limit sustainable energy consumption based on fossil fuel energy. A sustainable and effective way to meet these challenges is to explore renewable energy and develop energy storage technologies (including electric vehicle batteries).^1^ In recent years, lithium-ion batteries have been widely used in the fields of electric vehicles and renewable energy.^2–4^ Driven by the goal of “carbon neutralization”, the lithium-ion battery industry will usher in greater development space. Due to the advantages of high energy density and power density, ternary lithium-ion batteries occupy an important market in the field of global electric vehicles and electric energy storage.^5,6^ A ternary lithium-ion battery is a lithium-ion battery with a ternary material as the cathode electrode and graphite as the anode electrode.^7^ With the increasing requirements for energy density and mileage of power batteries, the cathode of a ternary lithium-ion battery is gradually developing in the direction of high nickel content. However, with the increase in nickel content, the cycle life and safety performance of a ternary lithium-ion battery are significantly decreased.^8^ The performance failure of ternary lithium-ion batteries has always been the focus of the industry.

Research on the performance failure of ternary lithium-ion batteries mainly focuses on the degradation of key materials, defects in the battery manufacturing process and abuse under different operating conditions.^9,10^ Researchers generally believe that the performance failure of ternary lithium-ion batteries is mainly caused by the deterioration in ternary cathode materials, including the replacement of lithium by nickel,^11^ the loss of cathode active materials,^12^ and materials rupturing and falling off during cycling.^13^ Research on the low-temperature performance failure of ternary lithium-ion batteries mainly focuses on lithium precipitation at the surface of the graphite anode electrode.^14^ The low-temperature environment will reduce the migration rate of lithium ions and promote the deposition of lithium ions on the surface of the anode electrode.^15,16^ The process of lithium deposition will consume active lithium, resulting in a reduction in the electrochemical performance and safety performance of the battery.^17,18^ A lot of work has been devoted to the study of lithium deposition mechanism,^19^ inhibition of lithium deposition^20^ and the impact of lithium deposition on battery safety.^21,22^ Through previous research, the lithium deposition mechanism has become clear,^23^ and some results have been achieved in inhibiting lithium deposition. However, there is still no fundamental solution to the problem of the low-temperature cycle performance failure of ternary batteries. The main reason is that the battery failure mechanism is described only from the level of cathode or anode electrode or electrolyte, but the three have not been combined and studied systematically. In addition, gassing is an important phenomenon of lithium-ion battery performance failure. Research on gassing of ternary lithium-ion batteries mainly focuses on cycling or shelving at room temperature^24^ and elevated temperature.^25^ The gassing mechanism is that the transition metal dissolves and catalyzes the decomposition of electrolyte at the anode interface.^24,26^ However there has been no report on gassing at low temperature, and lithium deposition at the anode interface will make the side reactions more complex.

This paper studies the performance failure phenomenon of ternary lithium-ion batteries under low-temperature operating conditions, and expounds the low-temperature cycle performance failure mechanism of the ternary lithium-ion battery under the synergistic action of cathode electrode, anode electrode and electrolyte. This study can provide a reference for the modification of the key materials of a ternary lithium-ion battery and the safe operation of a ternary battery.

## Experimental

In this experiment, a commercial 25 Ah ternary lithium ion battery is taken as the research sample. The cathode material is NCM532 (LiNi_0.5_Co_0.3_Mn_0.2_O_2_) and the anode electrode is graphite. The nominal voltage of the battery is 3.65 V and the standard charging and discharging current is 1C (25 A).

The cycling tester was a Neware BTS-5V50A. A Binder MKF115 high and low temperature environment test chamber was used for this experiment. After the temperature of the incubator reached the set temperature, the battery was put into an incubator and left for 4 hours, and then the cycling test started. The 1C current cycle performance of batteries at −10 °C and 25 °C was studied, leaving 5 minutes between each charge and discharge. The charge cut-off voltage was 4.15 V and the discharge cut-off voltage was 2.7 V. After the battery cycled at −10 °C, the battery was kept at 25 °C. After the battery inflated, a silica gel pad was pasted on the surface of the battery and a syringe pierced the silica gel pad to extract gas samples. Agilent gas-tight syringes (1 mL/10 μL) were used to sample 0.5 mL and washed with dry argon three times before each sampling. Gas components were analyzed with an Agilent 7890B GC. The gas chromatograph column was a HayeSep DB. The lithium evolution on the anode electrode surface of the new battery and the battery after low-temperature cycling was compared and analyzed. First the battery was discharged to 0% SOC, and then the battery was disassembled in a glove box filled with argon (water and oxygen content were less than 0.1 ppm). The cathode and anode plates were cleaned with dimethyl carbonate (DMC) and dried in the glove box for 12 hours. Finally, a sealed box was used to transfer the samples for the next step of analysis of physical and chemical characteristics.

The surface morphologies of the cathode and anode plates in different states were observed by a scanning electron microscope (SEM, FEI Nova NanoSEM 450). An X-ray diffraction instrument (D8, Bruker) was used to analyze the crystal structure of the battery cathode materials (*λ* = 0.154 nm). The composition and content of battery gas were analyzed (7890B, Agilent). The content of transition metal elements in the anode electrode material was analyzed (ICAP 6000, Thermo Scientific).

Transmission electron microscopy (TEM, FEI Tecnai G2 F30 S-TWIN) and X-ray photoelectron absorption spectroscopy (ESCALAB 250Xi, Thermo Scientific) were used to analyze the anode electrode. The changes in the anode electrode interface microstructure and chemical environment were studied.

## Results and discussion

### Electrochemical performance failure with low-temperature cycling

**Fig. 1:**
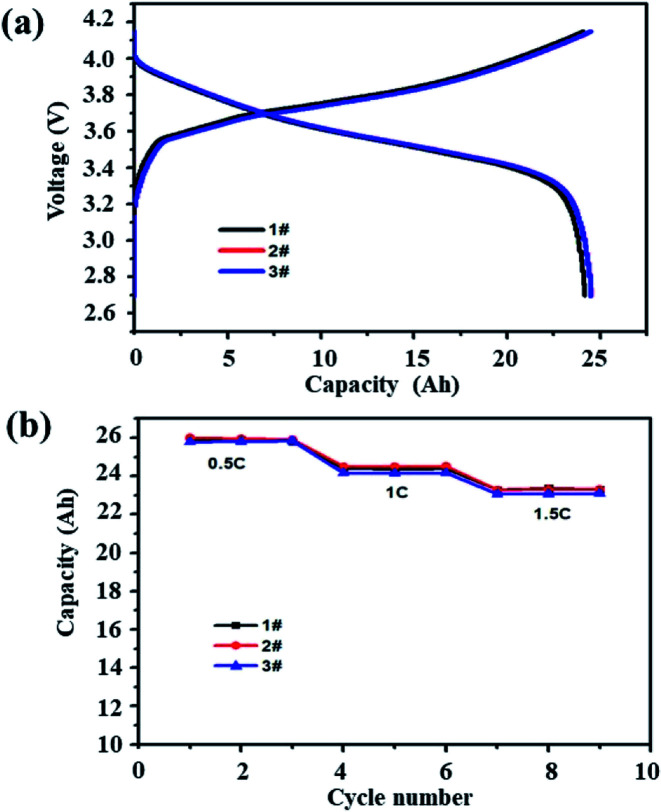
Three samples were randomly selected: (a) 1C charge–discharge curves and (b) discharge capacity with different currents.

Fig. 1 shows the capacity–voltage curves (a) of three battery samples under 1C current and the discharge capacity curves (b) under different currents. The curves of the three batteries basically overlap over the full voltage range. The average discharge capacities of the three batteries at 0.5C, 1C and 1.5C were 25.8 Ah, 24.5 Ah and 23.2 Ah, respectively. The deviation between the maximum capacity and the minimum capacity is within 1%, indicating that the consistency of the batteries is good.

**Fig. 2:**
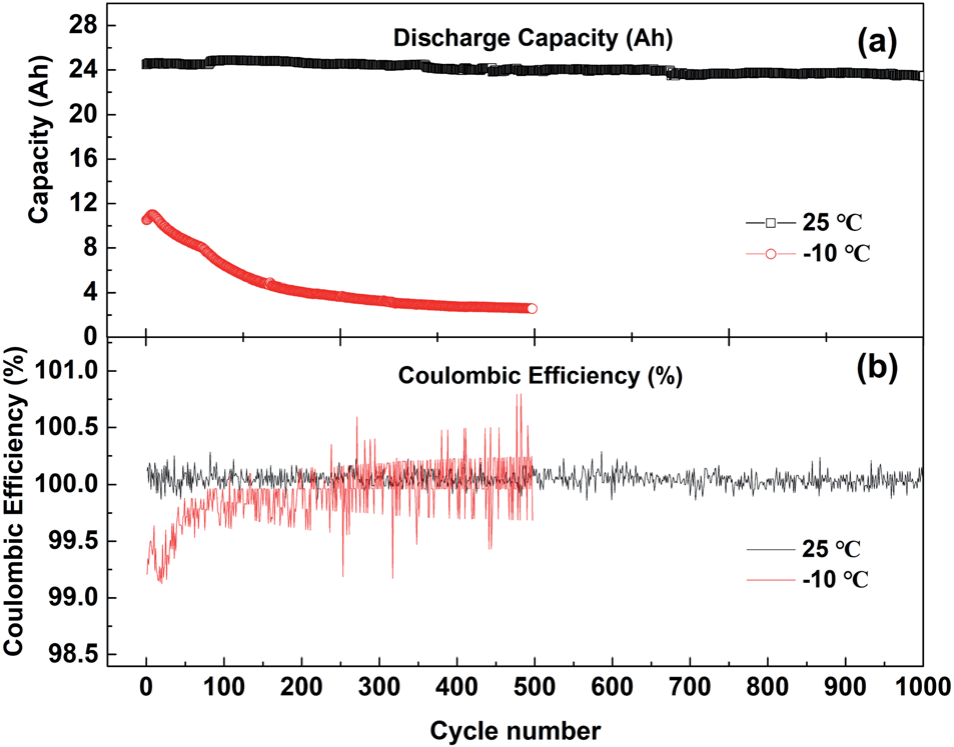
(a) The discharge capacity and (b) coulombic efficiency changes with the cycle number at 25 °C and −10 °C; the voltage range of charge–discharge is 2.7–4.15 V.

Fig. 2 shows the discharge capacity (a) and coulombic efficiency (b) curves of batteries cycling with 1C current at 25 °C and −10 °C. The voltage range of charge and discharge is 2.7–4.15 V. The discharge capacity of the battery cycling at 25 °C decreases slowly, from 24.5 Ah for the first cycle to 23.6 Ah after 1000 cycles, and the capacity attenuation rate is 3.7%. However, when the battery is cycled at −10 °C, the first discharge is only 10.5 Ah, and the capacity decreases rapidly. After 500 cycles, the discharge capacity is only 2.6 Ah. Even if the battery after low-temperature cycling is left at 25 °C for 4 h, then activated with 0.1C current cycling, the capacity of the battery can be restored to 18.3 Ah. But the low-temperature cycling still causes 25% irreversible capacity loss.

As shown in Fig. 2(b), the coulombic efficiency of the battery cycling at 25 °C is very stable, varying from 99.9% to 100.2%, while the coulombic efficiency of the battery cycling at −10 °C gradually increases from 99.2%. This indicates that during the charging process of the battery on the cathode side, the lithium ions removed from the cathode side are larger than those migrating back during the discharge. On the anode side, the lithium ions embedded in the charging process are larger than those removed during discharge. In this process, lithium ions in the cathode or electrolyte are continuously consumed and deposited on the surface of the anode. This may lead to the continuous accumulation of lithium ions in the anode electrode and the continuous reduction of active lithium atoms in the cathode electrode, resulting in the decline in low-temperature performance of the battery.

**Fig. 3:**
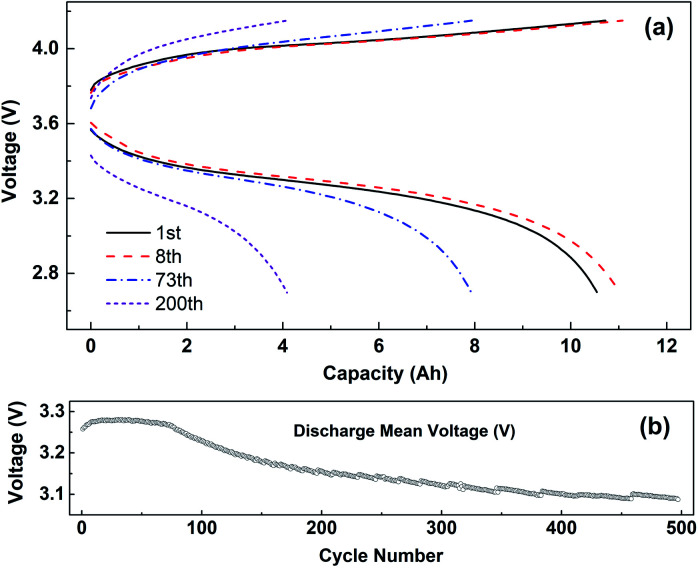
(a) The charge–discharge curves and (b) the variation curve of median voltage cycling with 1C current at −10 °C, and range of voltage 2.7–4.15 V.

Fig. 3 shows the charge–discharge curves (a) and the change curve of median voltage (b) of the battery cycling with 1C current at −10 °C. The voltage platform experienced a process of first decreasing and then increasing, which is caused by the polarization of the battery cycling at −10 °C. After 8 cycles of cyclic activation, the voltage platform decreased rapidly. The median voltage of the battery also experienced a change of first increasing and then decreasing. The median discharge voltage of the battery decreased from the initial 3.25 V to less than 3.1 V for 500 cycles.

**Fig. 4:**
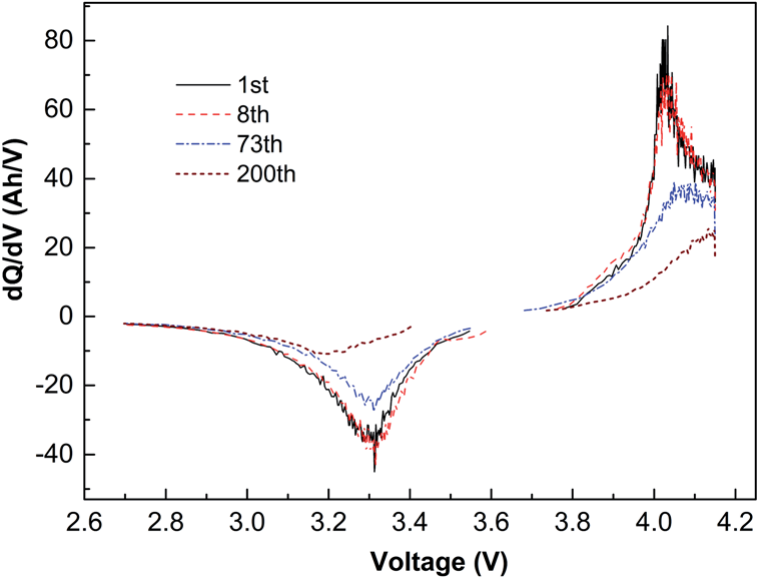
Capacity differential curves cycling with 1C at −10, and voltage 2.7–4.15 V.

Fig. 4 is the capacity differential curve of typical cycle times of the battery cycling at −10 °C with 1C current. The charging oxidation peak moves to the high-potential section, and the peak potential is 4.04 V. During discharging, the reduction potential moves to the low-potential direction, and the peak potential is 3.3 V. The charge–discharge potential difference of the battery is 0.74 V, which indicates that when the battery is cycling at low temperature, the migration rate of lithium-ion decreases, resulting in an increase in battery polarization.

**Fig. 5:**
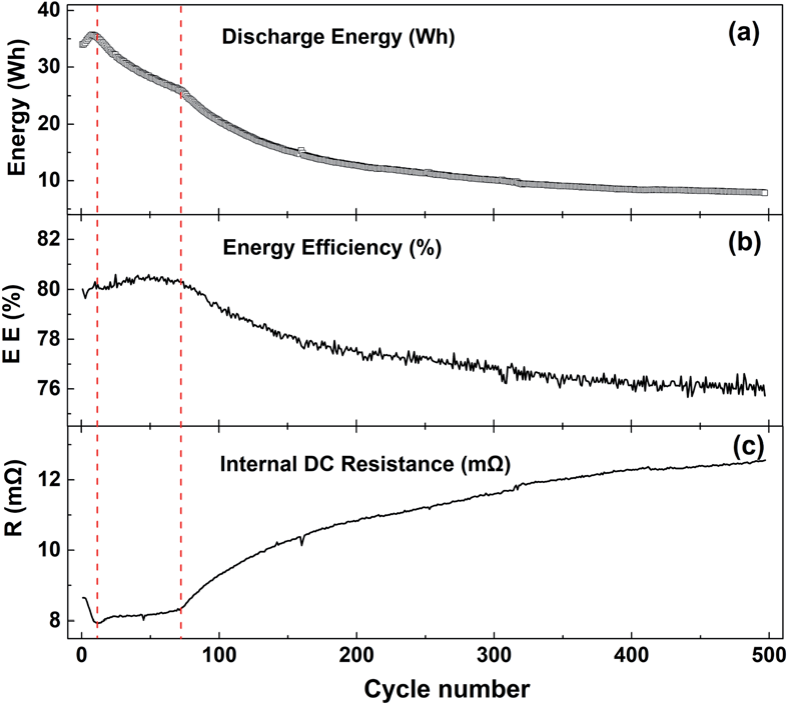
The curves of (a) discharge energy, (b) discharge energy efficiency and (c) full state DC internal resistance with cycle times at −10 °C at a current of 1C current over a voltage range of 2.7–4.15 V.

Fig. 5 shows the curves of discharge energy (a), discharge energy efficiency (b) and full charge DC internal resistance (c) with the number of cycles at −10 °C with 1C current. The whole cycle process can be divided into three stages. In the first stage, the battery discharge energy and efficiency rise rapidly, and the DC internal resistance decreases rapidly. The main reason is that the battery is in the activation stage. The second stage runs from the 8th cycle to the 73rd cycle. The discharge energy decreases rapidly, but the energy efficiency and internal resistance of the battery change little at this stage. The third stage is the accelerated decline in battery discharge energy and efficiency, and the rapid increase in DC internal resistance. Compared with the DC internal resistance of 1.1 mΩ cycling at 25 °C, the internal resistance at −10 °C is 8 mΩ, and the full current DC internal resistance after 500 cycles is as high as 12.2 mΩ.

### Battery gassing analysis

**Fig. 6:**
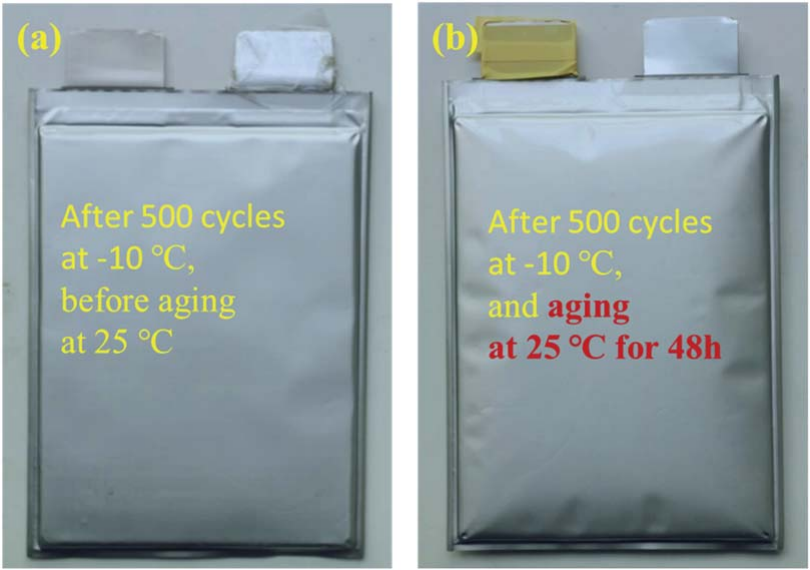
(a) Photograph of the battery after 500 cycles at −10 °C; (b) photograph of the battery stored for 48 h at 25 °C after −10 °C cycling.

The above analysis shows that low temperature will reduce the migration rate of lithium ions and increase the polarization of the battery. When the battery is cycling at low temperature, lithium deposition may occur at the anode electrode interface, which has a great impact on the life and safety of the battery. Fig. 6(a) is a photograph of the sample after 500 cycles at −10 °C and Fig. 6(b) is a photograph of the battery after 500 cycles at −10 °C then resting at 25 °C for 48 h. There is no obvious change in the appearance of the battery after low-temperature cycling compared with the new battery, but when the battery is put aside at 25 °C for 48 hours after low-temperature cycling, serious gas production occurs, which causes great potential safety hazards to the battery.

As shown in Table 1, the gas components are mainly H_2_, CO and CO_2_, and the other gas components are small molecular alkanes of C1–C3. Among them, the content of H_2_ is 32.61%, and the content of CO is more than that of CO_2_, which is quite different from the result that CO_2_ is the main component in the gas production by a ternary battery reported by Sascha Koch.^27^ It may be that in the process of gassing after a low-temperature cycle, the metal lithium deposited on the surface of the anode electrode participated in the gassing reaction, and some high-valence CO_2_ was reduced to low-valence CO.

**Table 1:** The percentage content of gassing components (%) after storage at 25 °C following cycling at −10 °C for 500 cycles

Gas composition	CO_2_	H_2_	CO	CH_4_	C_2_H_4_	C_2_H_6_	C_3_H_6_	C_3_H_8_
After 500 cycles at −10 °C	23.52	32.61	25.79	5.01	3.08	4.33	1.5	4.16

### Morphological and structural analysis

**Fig. 7:**
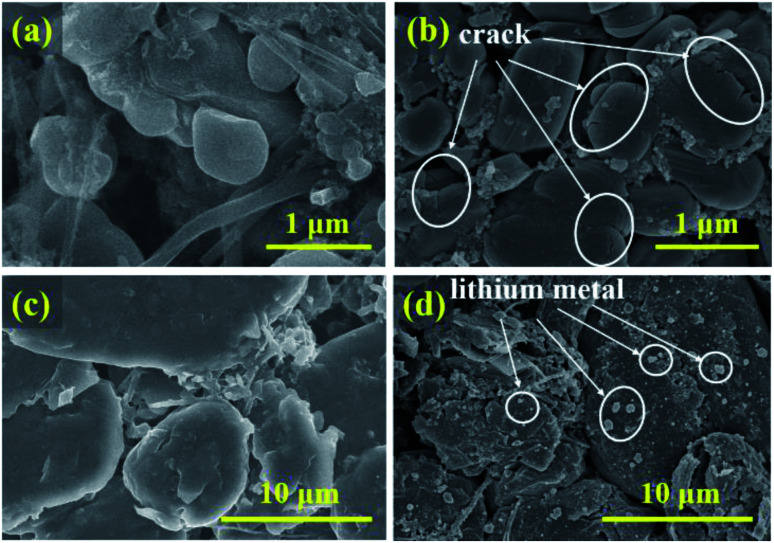
SEM images of the positive electrode of (a) the new battery and (b) after 500 cycles at −10 °C; SEM images of the negative electrode of (c) the new battery and (d) after 500 cycles at −10 °C.

To study the degradation mechanism of electrochemical and safety performance of a ternary battery under low-temperature cycling, the new battery and the battery after low-temperature cycling were disassembled in an argon atmosphere glove box. The morphology, structure and interface chemical environment of the cathode and anode electrode plates, as well as the components of battery gas were compared and analyzed. As shown in Fig. 7, compared with the SEM of the cathode of the new battery, after low-temperature cycling, the cathode particles show obvious cracking. The fragmentation of cathode material particles will lead to degradation in battery performance.^28,29^ White particles appear on the anode electrode surface after low-temperature cycling, which may be the metal lithium precipitated on the anode electrode surface.

**Fig. 8:**
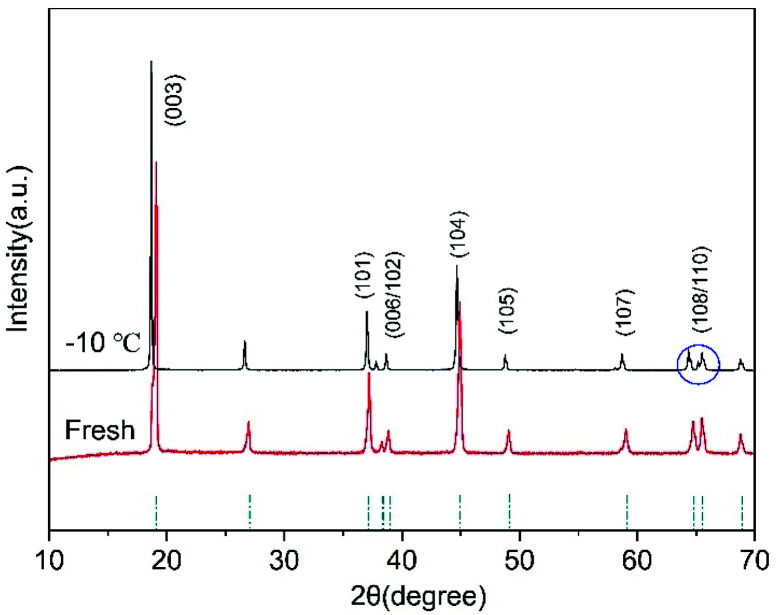
XRD of NCM after 500 cycles with 1C current at −10 °C.

After low-temperature cycling, the layered structure of the cathode electrode of the battery remains good, as shown in Fig. 8. However, the distance between the (108) peak and the (110) peak increases, and there is a small splitting phenomenon in the (110) peak. The splitting degree of (108/110) peaks is an important index for the ordering of the layered structure of cathode materials.^30^ Compared with the new battery, the layered structure of the cathode material deteriorated after low-temperature cycling.

### Interface analysis of the anode electrode

Since both lithium deposition and gassing side reactions occur at the anode interface, this section focuses on characterization and analysis of the anode interface.

**Fig. 9:**
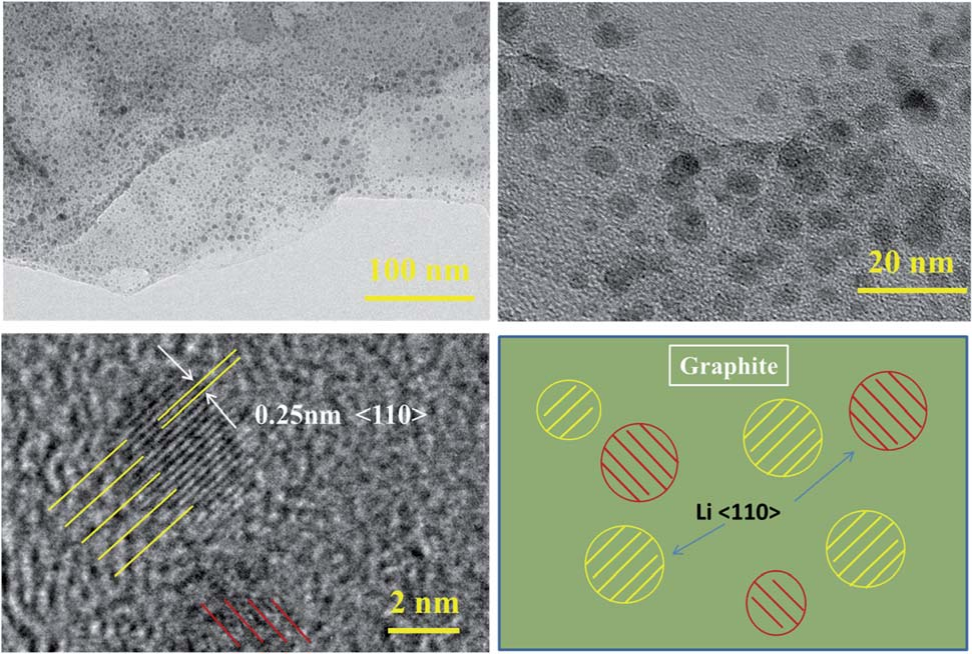
TEM images of the anode after 500 cycles with 1C current at −10 °C and a schematic diagram of lithium plating.

Many circular spots appear on the anode electrode surface of the battery after low-temperature cycling, and the diameter is mainly concentrated in the range of 1–5 nm, as shown in Fig. 9. It can be found from TEM that the spots have clear lattice stripes with a spacing of about 0.25 nm, which correspond to the crystal surface of metal lithium 〈110〉,^19^ indicating that serious metal lithium deposition occurs on the surface of anode graphite during low-temperature cycling.

**Fig. 10:**
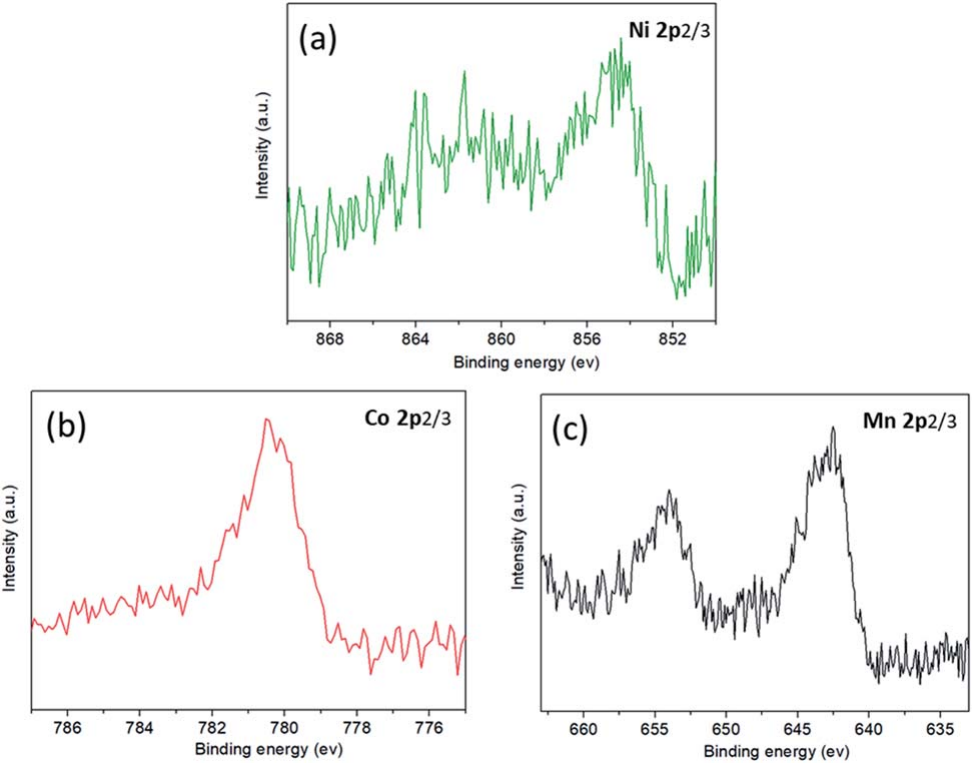
The XPS spectra of the anode surface after cycling at −10 °C: (a) Ni absorption spectrum; (b) Co absorption spectrum; (c) Mn absorption spectrum.

The analysis of anode interface elements is a common method to study anode interface side reactions. There are nickel, cobalt and manganese elements on the anode electrode surface after low-temperature cycling, as shown in Fig. 10. This shows that a small amount of transition metal atoms dissolve from the cathode electrode, then migrate to the anode electrode and precipitate on the surface of the anode electrode. According to the binding energy positions of nickel, cobalt and manganese XPS, it can be inferred that nickel, cobalt and manganese elements do not exist as simple substances in the anode electrode, but in the oxidation states of Ni^2+^/Ni^3+^, CO^2+^/CO^3+^ and Mn^4+^.^31–34^

After battery cycling at −10 °C, the ICP-AES analysis results of anode metal elements in the 0% SOC state are shown in Table 2. The contents of Ni, CO and Mn metal elements in the anode electrode of the battery after low-temperature cycling are 39 ppm, 29 ppm and 40 ppm, respectively, while those in the new battery are 7 ppm, 4 ppm and 15 ppm respectively, indicating that the content of transition metal elements in the anode electrode increases greatly after low-temperature cycling. There are two main mechanisms of transition metal dissolution. One is the HF corrosion mechanism,^35^ and the other is the defect mechanism of cathode materials.^36^ The fragmentation of cathode materials will accelerate the dissolution of transition metals.^28,29^ The cathode particles are broken in low-temperature cycling, which has been proved by the SEM in Fig. 7. This may be the reason for the dissolution of transition metals at low temperature. The whole process will also produce small changes in the cathode structure of the battery, which also confirms the analysis results of XRD.

**Table 2:** Contents of metal elements in the anode of the new battery and battery after cycling at −10 °C

Content of transition metal elements (ppm)	Ni	Co	Mn
New battery	7	4	15
After 500 cycles at −10 °C	39	29	40

### Low-temperature failure mechanism

**Fig. 11:**
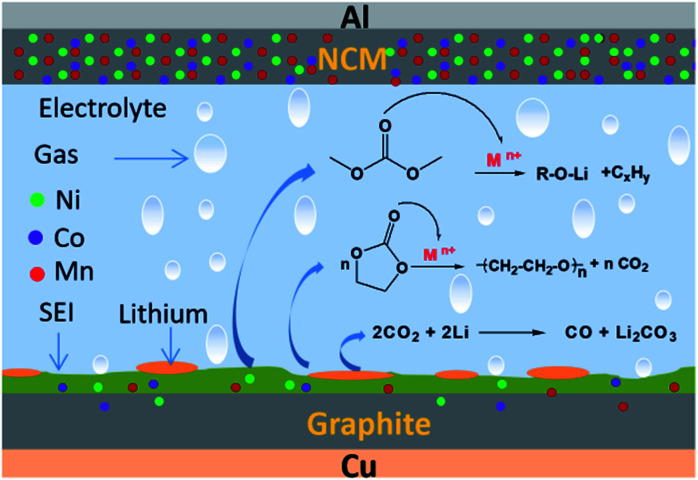
Schematic diagram of lithium plating and gassing mechanism after cycling at −10 °C.

According to Fig. 11, the low-temperature failure mechanism of a ternary lithium-ion battery includes the generation of “dead lithium” at the graphite anode electrode interface, and the dissolution, migration and deposition of transition metal ions in the cathode material. It also includes the process of gas production of electrolyte solvent catalyzed by transition metal ions after cycling at −10 °C.

When the reduction rate of lithium ions is greater than that of lithium atom embedded in graphite, they will be deposited on the surface of the anode electrode and lithium precipitation will occur.^37^ During the charging process of a ternary lithium-ion battery at low temperature, lithium precipitation occurs on the anode electrode surface. During the discharge process at low temperature, some of the precipitated metal lithium will dissolve, then oxidize into lithium ions and migrate back to the cathode electrode. However, not all the precipitated metal lithium will be oxidized into activated lithium ions, and metal lithium that loses direct contact with the anode electrode will lose electrochemical activity.^38^ The precipitation of metal lithium on the surface of the anode electrode and the formation of “dead lithium” are the main reasons for the decline in electrochemical performance, such as the reduction in low-temperature cycle capacity and the increase in internal resistance of a ternary battery.

**Fig. 12:**
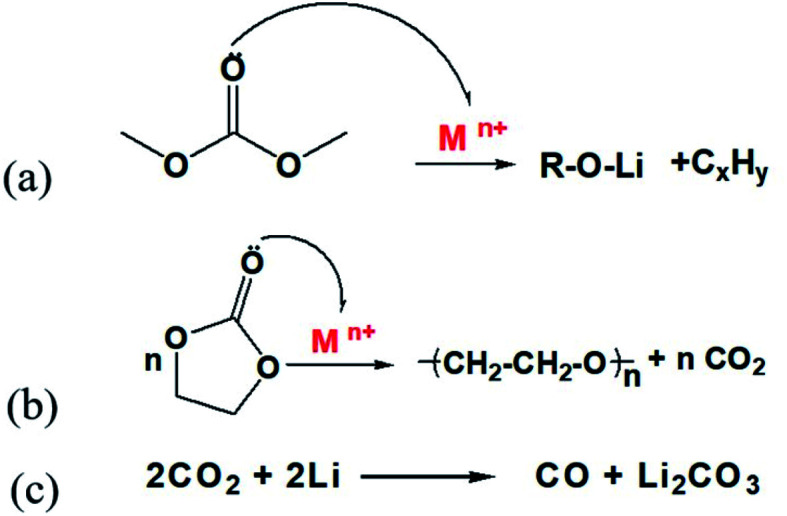
Chemical reactions involved in the proposed gassing scheme.

Combined with the results of XRD and XPS analysis, it can be inferred that a small amount of nickel, cobalt and manganese transition metal ions of the cathode material will dissolve during cycling, and pass through the diaphragm under the action of the electric field, then deposit on the surface of the anode electrode. The deposited transition metal ions show strong catalytic activity and can combine with the lone-pair electrons of the C=O bond in organic solvents. Cyclic organic solvents such as PC and EC can undergo ring opening, chain breaking and other reactions induced by transition metal ions M^*n*+^ (Ni^2+^/Ni^3+^, Co^2+^/Co^3+^ and Mn^4+^), resulting in a large amount of gas, as shown in Fig. 12.

When the battery was at low temperature, the side reaction of transition metal ion catalytic decomposition of electrolyte solvent was difficult. However, after low-temperature cycling, the battery was kept at room temperature. With an increase in ambient temperature, the catalytic reaction of gassing took place rapidly. In addition, the “dead lithium” on the anode electrode surface has very active chemical properties. At an appropriate temperature, the organic solvent or CO_2_ in the battery can also be reduced to CO. This is the main reason for gassing of a ternary battery during normal temperature storage after low-temperature cycling.

The low-temperature performance failure of a ternary lithium-ion battery includes side reaction processes such as anode metal lithium deposition, dissolution of cathode material transition metal, and electrolyte decomposition. The side reactions are interrelated and synergistic, and jointly accelerate the performance decline of ternary lithium-ion batteries. Inhibiting lithium evolution and reducing the generation of “dead lithium” is the key to improving the low-temperature cycling electrochemistry of ternary lithium-ion batteries. Reducing the dissolution of transition metal ions from cathode materials through surface modification is an effective way to reduce gassing and improve the safe operation of ternary lithium-ion batteries.

## Conclusions

The failure of the electrochemical performance of a commercial 25 Ah ternary lithium ion battery cycling at −10 °C was analysed by coupling internal and external characteristics. The increase in DC internal resistance is the main reason for lithium plating. Dead lithium at the anode interface leads to irreversible reduction in the capacity of the battery at low temperature. The deposition of the cathode transition metal ions at the anode interface and catalytic decomposition of the electrolyte are the main reasons for gassing. Lithium precipitation participates in and accelerates the gas production process. The failure of the low-temperature performance of a ternary lithium-ion battery is the result of side reactions such as lithium deposition, dissolution of cathode material transition metal ion and electrolyte decomposition. Inhibiting the formation of lithium plating is the key to improving the low-temperature electrochemical performance of a ternary lithium-ion battery. Reducing the dissolution of transition metal ions of cathode material is an effective way to reduce the gas production of a ternary lithium-ion battery.

## Conflicts of interest

There are no conflicts to declare.

## Supplementary Material
